# Supplementary material for: Bax assembles into large ring‐like structures remodeling the mitochondrial outer membrane in apoptosis
Source: EMBO J. 2016 Jan 18;35(4):402–13. doi: 10.15252/embj.201592789 (PMC4755111; doi:10.15252/embj.201592789)

Appendix

Figure S1 - Bax clusters often appeared not to be fully embedded into the outer membrane but to be attached to the mitochondrial surface.

Dual-color STED nanoscopy of mitochondria in apoptotic U2OS cells labeled with antibodies against the mitochondrial outer membrane protein Tom20 (red) and Bax (green). The arrows point to Bax clusters. Numerous Bax clusters on the mitochondrial membrane appear not to be embedded in the membrane, but to be attached with only a fraction of the cluster extending in the outer membrane. Scale bar: 1 µm.

Figure S2 - The area encircled by a Bax-ring is devoid of the mitochondrial outer membrane protein Sam50 and Bax-rings form independently of Z-VAD-FMK.

A Mitochondria of apoptotic U2OS cells were decorated with antibodies against Sam50 (red) and Bax (green), as indicated. The Bax signal was recorded in the STED mode. Shown are the individual color channels as well as an overlay. Scale bar: 0.5 µm.

B Mitochondria of apoptotic U2OS cells, which were not treated with Z-VAD-FMK and decorated with antibodies against Tom22 and Bax. Otherwise imaging as in (A). Lagrange interpolation was used to double the number of pixels. Scale bar: 0.5 µm.

Figure S3 - The release of cytochrome *c*, but not of Smac/DIABLO is delayed in Drp1 knockdown cells.

A, B, C Shown are confocal recordings of Drp1 knockdown U2OS cells 14 h after induction of apoptosis by actinomycin D. The cells were labeled for Bax and Smac/DIABLO (**A**), for Bax and cytochrome *c* (**B**), and Smac/DIABLO and cytochrome *c* (**C**). The arrows point to cells with activated Bax. The asterisks mark cells which exhibit a mitochondrial cytochrome *c* signal, but no mitochondrial Smac/DIABLO signal, indicating that the mitochondria in these cells have already released Smac/DIABLO but not yet cytochrome *c*. Scale bar: 50 µm.

Figure S4 - The redistribution of the MICOS core components Mic27 and Mic60 precedes the release of cytochrome *c* in apoptotic Drp1 knockdown cells.

A, B The mitochondria of unchallenged Drp1 knockdown cells (DMSO) and of apoptotic Drp1 knockdown cells (pre-release and post-release) were decorated with antibodies against Mic60, Bax and cytochrome *c* (**A**) or with antibodies against Mic27, Bax and cytochrome *c* (**B**), respectively. The cytochrome *c* signal was used to classify the apoptotic state of the mitochondria as pre-cytochrome *c*-release (pre-release) and post-cytochrome *c*-release (post-release). In the mitochondria of unchallenged cells, Mic60 (**A**) and Mic27 (**B**) are localized in distinct clusters. In apoptotic mitochondria, indicated by the activation of Bax, the fluorescence signals of both MICOS proteins is rather uniformly distributed, independent of the cytochrome *c*-status. The pixel numbers of the recorded images were doubled using Lagrange interpolation. Scale bars: 1 µm.

Figure S1

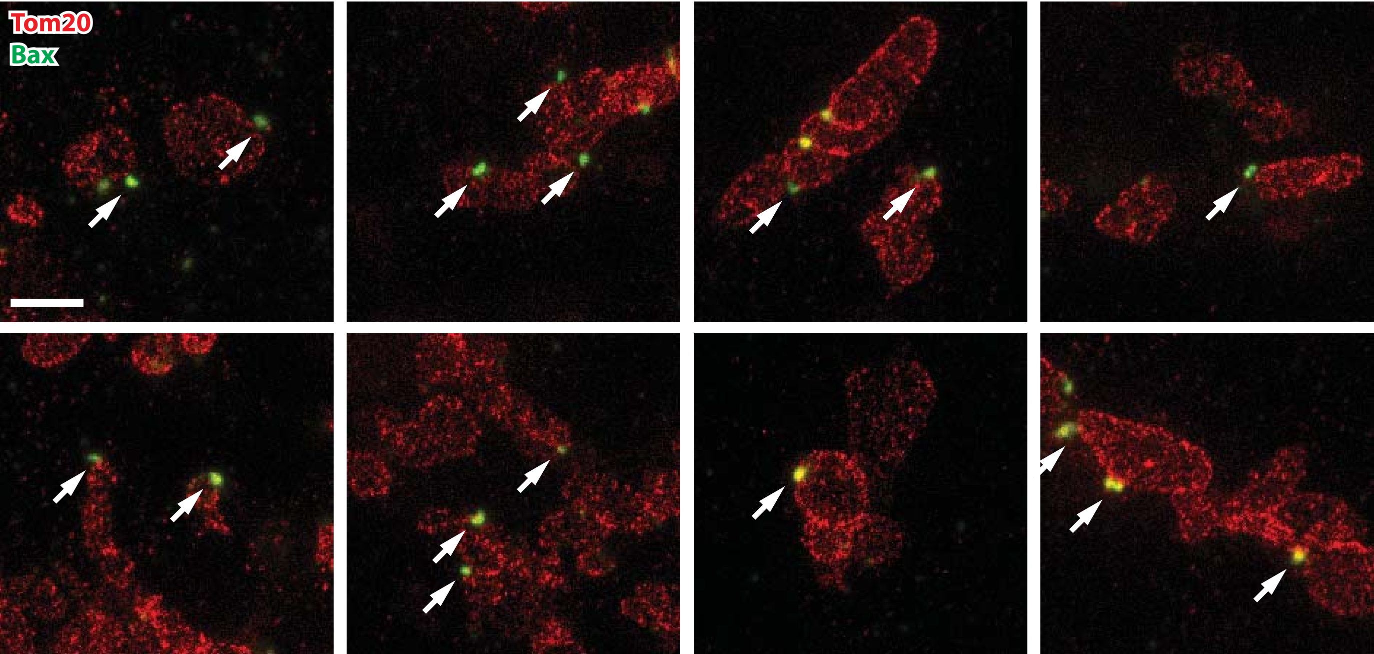

Figure S2

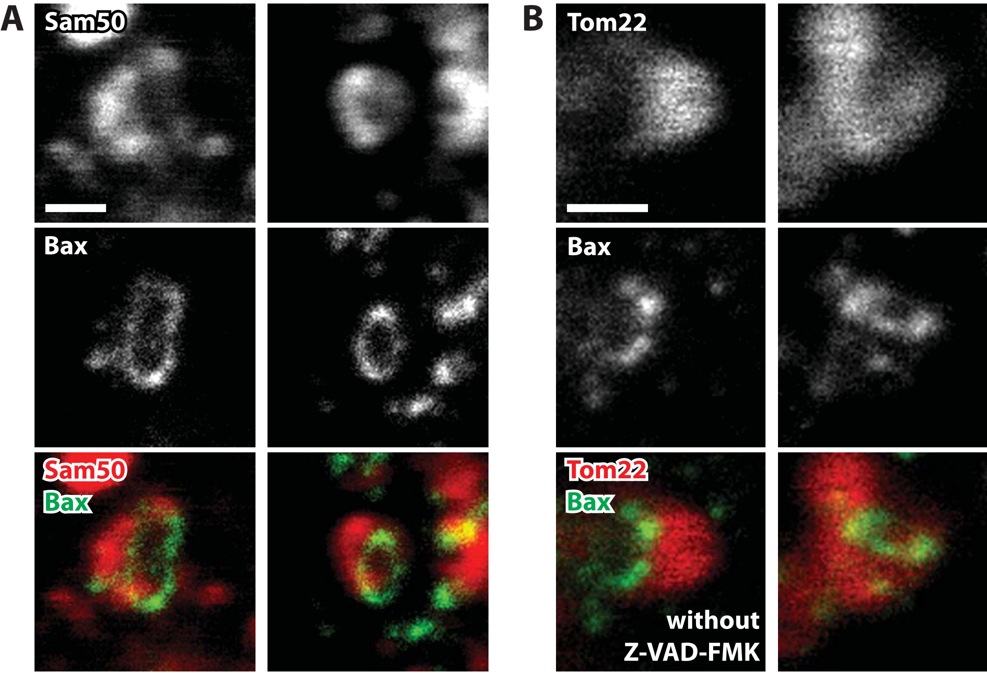

Figure S3

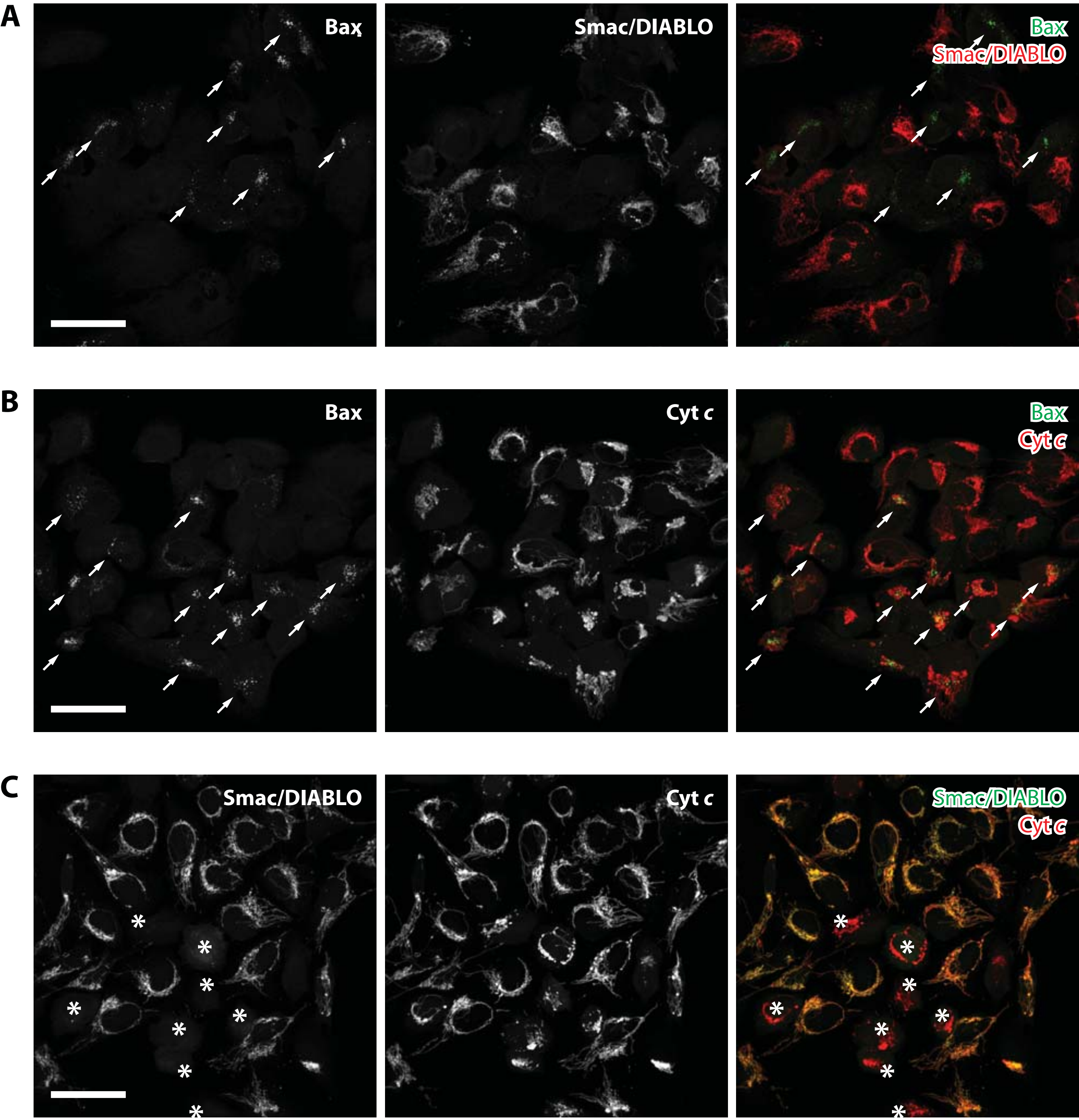
A
Bax
Smac/DIABLO
Bax
Smac/DIABLO
B
Bax
Cyt c
Bax
Cyt c
C
Smac/DIABLO
Cyt c
Smac/DIABLO
Cyt c

Figure S4

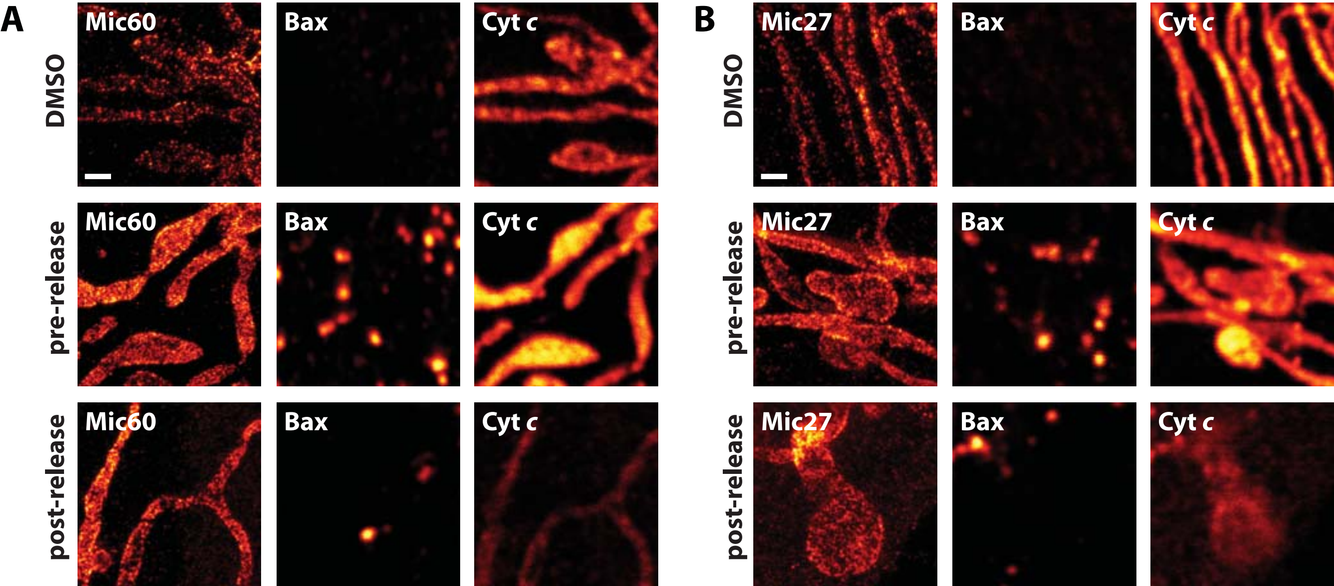

Supplement: Supplementary file 1 — Appendix [file EMBJ-35-402-s001.pdf]
